# Exploring the Feasibility and Potential of Virtual Panels for Soliciting Feedback on Nutrition Education Materials: A Proof-of-Concept Study

**DOI:** 10.2196/publichealth.5134

**Published:** 2016-04-19

**Authors:** Cameron D Norman, Helen Haresign, Christine Mehling, Honey Bloomberg

**Affiliations:** ^1^ Cense Research + Design Toronto, ON Canada; ^2^ Social & Behavioural Sciences Division Dalla Lana School of Public Health University of Toronto Toronto, ON Canada; ^3^ Dietitians of Canada Toronto, ON Canada

**Keywords:** health communications, Facebook, marketing, nutrition, nutrition education, healthy eating, health education, design, study design, social media, user design, qualitative data, health promotion, public health

## Abstract

**Background:**

A changing and cluttered information landscape has put pressure on health organizations to produce consumer information materials that are not only factual but high quality and engaging to audiences. User-centered design methods can be useful in obtaining feedback from consumers; however, they are labor intensive and slow, which is not responsive to the fast-paced communication landscape influenced by social media. EatRight Ontario (ERO), a provincial nutrition and health support program of Dietitians of Canada, develops evidence-based resources for consumers and sought to increase user-centered design activities by exploring whether the standard approach to feedback could be replicated online. While online feedback has been used in marketing research, few examples are available in health promotion and public health to guide programming and policy.

**Objective:**

This study compared a traditional in-person approach for recruitment and feedback using paper surveys with an Internet-based approach using Facebook as a recruitment tool and collecting user feedback via the Web. The purpose of the proof-of-concept study was to explore the feasibility of the approach and compare an online versus traditional approach in terms of recruitment issues and response.

**Methods:**

An exploratory, two-group comparative trial was conducted using a convenience and purposive sampling. Participants reviewed a handout on healthy eating and then completed an 18-item survey with both forced-choice items and open-ended responses. One group viewed a hard-copy prototype and completed a paper survey and the other viewed a PDF prototype via Web links and completed a Web survey. The total days required to fulfill the sample for each group were used as the primary method of efficiency calculation.

**Results:**

In total, 44 participants (22 per condition) completed the study, consisting of 42 women and 2 men over the age of 18. Few significant differences were detected between the groups. Statistically significant (*P*≤.05) differences were detected on four attitudinal variables related to the document reviewed and include perceived length of the document, perceived attractiveness, likelihood of contacting ERO for food and nutrition questions in the future, and likelihood of recommending ERO to a friend. In all cases, the responses were more favorable to the document or ERO with the online group. All other variables showed no difference between them. A content review of the qualitative feedback found relative consistency in word use and number of words used, indicating relative parity in the amount of data generated between conditions. The online condition achieved its sampling target in 9 days, while the in-person method took 79 days to achieve the target.

**Conclusions:**

An online process of recruitment through Facebook and solicitation of online feedback is a feasible model that yields comparable response levels to in-person methods for user feedback. The online approach appears to be a faster and less resource-intensive approach than traditional in-person methods for feedback generation.

## Introduction

A diverse and cluttered informational environment has placed pressure on health organizations to devise means to communicate with their audiences that attract attention to their messages and provide relevant advice. Social media has further complicated this challenge, introducing new opportunities and demands on organizations operating with limited resources as they try to attract an audience that is presented with increasing competition for its attention [1]. Attracting and holding consumer attention and engaging audiences requires strategic design considerations that differ from traditional health communications messaging where information flows are unidirectional, not multidirectional as is the case with social media. This new participatory media landscape requires attention to information quality and the health and eHealth literacy of audiences, but also to their preferences, interests, and the esthetic appeal of communications that take place within the media ecology [2]. Thus, it is critical that health organizations develop materials that take into account user preferences and interests and do so in a manner that is responsive, proactive, and consistent with the rapid media cycle available through social media. This study seeks to explore how social media can be used as a means of providing reliable consumer feedback on health promotion and nutrition education materials in an efficient manner compared with traditional face-to-face consultations with users.

### Background

For health organizations, the requirements and expectations around their communications activities are growing amid limited resources (eg, time, money), which places ever-greater emphasis on the need to be efficient and effective in health communications campaigns. This requires attention to the needs and use contexts of the audience in the co-development of the message and determining the media forms that are best suited to delivering, exchanging, and co-creating messages [3]. User-centered design is an approach that seeks to create products and services that are based on the preferences, needs, and use patterns of the intended audience (users) and creates more relevant and potentially more used products and services [4]. While potentially helpful, such design methods are time and resource intensive, especially for organizations that serve broadly dispersed populations.

One way to gain user feedback in structured form is the virtual panel, which is a growing staple within the marketing profession [5]. Virtual panels go by many names (eg, customer advisory panels, online research panels, Internet access panels), but they typically are standing groups made up of recruited participants or volunteers who are typically called on to provide feedback on specific things over time [5]. Virtual panels and online surveys conceptually offer many advantages for health promotion. Virtual panels reach those who are not seeking support from a professional (eg dietitian), are not limited by geography in the same way, potentially reach a greater diversity of individuals, and may be a cost-effective means of nutrition education and research [6-8]. Social media adds to the opportunity for recruitment of participants and deployment of virtual panels for soliciting feedback on materials or engaging the public in health promotion campaigns [8-13].

### Organizational Context and Setting

EatRight Ontario (ERO) is a province-wide program designed to provide residents of Ontario with increased access to evidence-based healthy eating information, educational outreach, and consultation through a variety of distance-bridging methods. ERO is a multiplatform free service providing dietitian and healthy eating support services to residents of Ontario through printed materials, a toll-free telephone dietitian advice line, email-a-dietitian service, Web-based resources, and social media. The ERO service is operated by Dietitians of Canada (DC) with funding from the Ontario Ministry of Health and Long Term Care. The materials and advice offered by ERO are developed by DC as part of the Practice-based Evidence in Nutrition (PEN) database and include a variety of modalities and media accessible through the ERO website, social media, phone, email, and mail.

At the time of this study’s deployment, the standard protocol for feedback gathering involved sharing early prototypes of materials under development to professional dietitians who volunteered to consult with their clients about various features related to the length, attractiveness, and perceived quality and usability of the materials. This approach required that a designated coordinator recruit volunteers, collect contact information from them, prepare and distribute materials in hard-copy form by mail to volunteers, send reminders, collect returned surveys or feedback forms completed by clients, the volunteers or both, and then manually collate the materials. This was perceived to be slow, inefficient, and potentially prone to error.

This study seeks to consider the differences associated with using a virtual panel approach to recruitment and deployment of a user feedback process compared with the standard in-person approach currently in use. The in-person approach has consisted of sending draft materials to dietitians in the field for feedback and who may also solicit feedback from among their clients. A switch to using online recruitment and feedback gathering holds the potential to improve the reach, speed, and potential to do more iterative reviews if found to be comparable to the current standard practice in the quality and quantity of feedback received. This proof-of-concept study seeks to explore this issue and provide guidance for future research.

### Project Outline

The overall project included three components: (1) a review of the literature on virtual panels as a means of feedback elicitation and design critique of consumer-directed materials, (2) a review of the program options and developmental design of the intervention (ie, the means of soliciting feedback through online methods), and (3) a comparative experimental study exploring the outcomes of two methods of soliciting feedback. The results of the comparative analysis are reported here. The study employed a collaborative, participatory, and co-creative process between ERO, PEN, and the lead researcher (CN) reflecting the co-creative process that was of interest in the study. This enabled participation on the development of measurement items, outcome indicators, and in the sense-making process required to interpret the findings.

Social media offers a real-time manner of recruiting participants and soliciting feedback from potential users of materials under review, particularly because it can engage them directly, and their engagement might have ancillary benefits beyond the current project by having them associate with DC and ERO as brands. ERO has a broad and engaged following among the public and professionals alike through social media. The online approach to engagement was selected and designed based on an initial review of the current ERO online portfolio, to assess which of the three would be the most feasible option to start with. All three of the social media platforms have active users/followers. However, among the various options Facebook was selected as the medium of choice to begin the study.

ERO has extensive experience with using social media as communication tool and as a vehicle to try new means of engaging their audience through different media. As of September 2015, ERO’s social media properties included 9261 “likes” on Facebook, 2338 subscribers to their YouTube channel (with multiple videos having been viewed more than 100,000 times and over 700,000 total views of all videos) and 9647 followers to their Twitter feed and over 15,000 subscribers to a newsletter that has used social media as a recruitment tool for attracting enrollment.

In reviewing the various options, the community that had formed through ERO’s Facebook page was highly active with many different users posting responses to ERO’s posts, questions, and events, suggesting it might be the best place to design and trial an engagement plan for recruitment of a panel. A separate FB group associated with ERO’s page was established, and invitations were sent out to all page members (ie, those who had “liked” the page). Based on the study design, membership in this special group was capped at 24 people. Anyone who requested inclusion in the group up to this number was included; no selective recruitment was conducted. In considering future possibilities for ERO, Facebook also offered the most opportunity to develop and maintain a panel over time compared to other forms of social media engagement (eg, Twitter).

The study protocol received approval from the University of Toronto research ethics board (Protocol #00029006), and no adverse effects were reported during the study.

## Methods

An exploratory, two-group comparative trial was conducted using a convenience and purposive sampling comparing the current model of feedback used by DC and the ERO service with a novel, online approach. In the first condition, the current standard approach for feedback elicitation was used. DC sent a message to its members requesting assistance with the study. Those dietitians who agreed to participate were to ask clients post-consultation if they would be interested in participating in the study, and those who indicated an interest were given the consent and information package to review. Consenting individuals were given a survey and hard-copy version of the information resource under review entitled *Healthy Eating Guidelines for Increasing Your Fibre Intake* (Multimedia Appendix 1) and completed the survey in a private space, sealed it in an envelope, and returned it to their dietitian to return to DC. This is consistent with the previous standard practice except that feedback was normally provided orally to the dietitian and the responses were not blinded to the dietitian. In this case, responses were blinded, which was more consistent with a true study and helped mitigate social desirability bias.

In the second condition, a request for participation message was posted on the ERO Facebook page with instructions for interested individuals to contact the ERO communications manager who was responsible for posting material to the Facebook page on behalf of ERO. Once the desired quota of 24 participants was reached, recruitment was closed. All interested participants were invited to a special subgroup of the Facebook page that was designed for the study and restricted as invite-only for the purposes of the study. A link to an electronic Web survey hosted on FluidSurveys was provided that had a preamble with the consent materials and an embedded link that opened up an electronic version of the document *Healthy Eating Guidelines for Increasing Your Fibre Intake* for users to review online*.* In both conditions, participants were given an opportunity to indicate if they wished to receive a complimentary Dietitians of Canada cookbook in acknowledgment of their participation.

### Materials

An 18-item, self-administered survey that asked questions about usability, esthetics, health behavior, and demographic questions was developed for the study drawing on some of the questions used in past practice by DC in their previous resource reviews. The survey included the ethical consent information as a preamble, which allowed individuals to indicate their interest in participation and gain an understanding of the risks and benefits prior to participating in the study (Multimedia Appendix 2). The survey was not pre-tested and did not undergo any psychometric assessment due to its short size and straightforward opinion questions with open-ended responses. The online version of the survey was restricted to only those with a secure Web link to the Fluid Survey platform.

### Recruitment

48 participants—24 in each condition—were recruited between February and April 2014 to participate in the study. A minimum sample size of 20 participants per condition was sought as the required number in this efficacy trial. To account for possible dropouts, the study oversampled with 48 initial recruited participants, of which 44 eventually completed the study with 4 who left the study prior to completion (2 per condition). Participants were recruited by two means: (1) standard practice through dietitians operating in Ontario via an in-person introduction to the study after the client has completed their appointment or (2) an open invitation to join a research study panel subgroup on the ERO Facebook page sent from the ERO communications manager. In both circumstances, individuals were presented with an invitation letter (in person or via a secure, confidential channel such as an email address) that introduced the study, its goals, its risks and benefits, procedures, and appropriate contact information with instructions to indicate interest and consent to participate. If interest was indicated (verbally to the dietitian or via an affirmative response through email or through direct Facebook message), a formal consent form was presented using the appropriate media and participants either signed the form and returned it to the dietitian (Condition 1) or selected a check box on the Web form (Condition 2).

The study approach followed a model of exploration and testing used within innovation research that uses a concept called design thinking [14], whereby initial ideas are generated, then refined and the most plausible, trialable product is tested with rapid feedback to allow for adaptation of the design as necessary. This approach is aligned with Developmental Evaluation [9], where the evaluation design is tied to the intervention and co-developed to ensure appropriate fit, scope, and adaptability to suit context. The use of Facebook as the medium for recruitment and deployment of the study was seen as the first choice among different options, but ended up being the appropriate choice after testing (ie, there was a positive response that yielded the level of engagement desired from participants), so no alternative methods were sought as the full sample was recruited.

In both conditions, participants were offered a Dietitians of Canada cookbook from ERO as a form of recognition for their time and were asked to submit an email to the ERO communications manager requesting one if they wished.

### Analytic Approach

Due to the small sample size and given the focus on efficacy and plausibility of the intervention (online vs standard forms of feedback), non-parametric tests (Mann-Whitney U) were conducted to determine any potential between-group differences. Standard demographic frequency calculations were used to collate the responses.

Qualitative comparisons were made using a content analysis to assess if there were any unique features of terms, language, or descriptive depth to the text. A word count was performed to see if there was any difference between the two groups.

### Efficiency Considerations

Data collection was tracked to reflect the overall speed of delivery and response. Post-hoc review of coordination time spent on the study was used to determine amount of staff energy used to facilitate the study.

## Results

Forty-four participants completed all aspects of the study, 22 in each condition with 2 participants who did not complete the entire survey; 42 women (95%) and 2 men (5%) completed the study. Age was calculated by range response (20 years per category), with all participants reporting falling between 19 and 70 years, with the mean age range of 31-50. No participants reported being under the age of 18 or over age 70. Differences between groups were detected on age and sex, with no men participating in the online group and a slightly higher mean age for those in the paper condition.

### Quantitative Results

We conducted Mann-Whitney U comparisons to explore differences between each group on each of the variables using IBM SPSS Statistics 22.

Statistically significant (*P*≤=.05) differences were detected on four attitudinal variables related to the document reviewed and include perceived length of the document (ie, number of pages) (*P*=.027), perceived attractiveness (*P*=.022), likelihood of contacting ERO for food and nutrition questions in the future (*P*=.029), and likelihood of recommending ERO to a friend (*P*=.002). Across all questions, the online group reported more favorable responses to questions than those in the in-person, paper-based condition. All other variables showed no comparative difference between them.

### Qualitative Results

No discernable style difference was found in comparing the responses between the two groups across the three open-ended questions. Three open-ended questions were asked on the survey: (1) A supplemental item to Q3 requesting additional comments on the look of the handout, (2) “After reading the handout, tell us 1-3 changes that you want to make in your diet?”, and (3) “What would make this handout more useful to you?”

Some of the examples of the quote comparisons are included in Tables 1-3. Qualitative text is taken directly from the survey and has been edited only for formatting, not grammar. Table 1 presents some of the responses to the question, “What would make this handout more useful to you?”

With the first response, the total word count was 368 words across 10 responses to the item, (36.8 words per response, 45% question completion) with the online survey, compared with 321 words used across 13 respondents (24.7 words per response, 59% question completion) to the item in the paper survey. All other participants did not provide an answer to the question.

Table 2 profiles some of the responses to the item, “After reading the handout, tell us 1-3 changes that you want to make in your diet.”

**Table 1:** Usefulness of the handout: selected responses.

Online group	Paper group
I’m more of a visual type. I feel that if the steps to eating more fibre would be easier to retain and refer to if it was in a table type format. It would be great if you could get the meal plan to fit on one page. That way, people could easily pin it up or put it on their refrigerator.	I like the use of bulleted points and tables. As something you can print out from the Internet this is great. However, it looks like the formatting of this print handout has been done using HTML. The look is not at all optimal for a printed document in terms of layout, page breaks, and visual separation of elements. Double borders on a table looks terrible!
It is very clear and concise. Easy to read. Easy to use as a guide. One comment on the comparative table re: high fibre/low fibre diet. A couple of places have the same food item at the meal but they don’t line up on either side.	Consider bolding headings to standout (eg, pg. 2 veg, fruit, legumes, nuts and seeds) - add pictures of food if possible - consider hyperlinks to move info on a certain food that someone might not know about eg, guava? - chart (meal breakdown) is good/helpful to see - more spacing between groups (topics) so they standout
Most people won’t read past the first page.	To think about > - colour - print double sided on 17 x 11 P - fold - then it’s just one piece of P
	1 - Good layout - PEN logo busy at the top (simplify logos > distracting) - maybe have choice of font sizes? (large for older people, medium for younger > you can then have better spacing for blocks + info)
I think it is a great handout	A few colours or highlighted areas - numbers instead of words

**Table 2:** Anticipated or intended behavior changes attributable to the handout: selected responses.

Online group	Paper group
Change my cereal, use a combination of flour and include more fibre in my snacks. Flax seed to my smoothie	I want to be more aware and make sure I am including the right foods to obtain my daily fibre since I deal with IBS symptoms
Add more fibre easily by swapping current food choices for those with a higher fibre content.	Instead of having juice at breakfast to have an orange or other piece of fruit with my cereal 2. to try and have oatmeal more
The handout listed a lot of the changes that we are currently making in our diet. I have not thought of using dried fruit as a snack. That is easy to transport and store. I will make that change. And I have not thought of added beans to a pasta sauce. I’m going to try that as well.	add + look for more variety in choices/meals - try to changes to recipes (substitutions to the every day) - read more labels
Simple substitutes for the foods I already eat for ones with more fibre - Chart was very helpful - Will make sure I am get “whole grains” not just “whole wheat”	Be more fearless in substituting 1/2 to full white flour to a multi-grain. Reference fibre counts. Get back on your Eat Right Ontario website to see the latest healthy recipes (especially using legumes)

For this question there was a difference in the number of words used with participants in the online group writing a total of 484 words (mean of 24.2 per response, 91% overall completion) and those in the paper group writing a total of 331 words (mean of 15 per response, 100% overall completion).

Table 3 highlights some of the comments made to the question, “What would make this handout more useful to you?”

**Table 3:** Usefulness suggestion responses.

Online group	Paper group
Make available on line	an extensive listing of foods and their gm of fibre. Fruits and vegetables
I recently learned that you can increase the amount of fibre intake all you want but it is not productive in your system if you are not drinking enough water. I think that should be stated somewhere or highlighted as an important factor esp for people using this type of diet to have more regular bowel movements. Water helps break down the fibre. It would be nice to have a link to recipes that include high fibre ingredients such as homemade granola bars, quinoa salad, etc	shorter to read - more point form - pictures, graphics + charts
You are on Facebook that is a big help for all that want the help.	if the information was condensed. Somehow to make the handout shorter > maybe 4 pages. more websites in the additional resource section to get more ideas on how to add more fibre to my diet - have more examples that are culturally sensitive if possible - more examples of fruits/vegetables with high in fibre
I would like to see more suggestions on how to make food substitutions! if you eat this now... here is what you can try to replace it with for more fibre.	Take out the 2-page chart, it didn’t add much. Add a good recipe.

The two groups differed marginally on the completion rate, with the online group yielding 12 responses (55% item completion) and the paper group yielding 8 responses to the question (36% item completion). For word count, the online group overall number of words was lower with 205 (mean of 17 words per response) compared with the paper group with 245 words (mean of 30 words per response).

### Efficiency Results

The ability to reach the quota of 22 participants (per condition) reflected a substantive difference between the two groups. The recruitment for both arms of the study began at the same time. For the in-person group, this began with emails and phone calls placed to dietitians requesting their assistance, which was consistent with the current practice. In the online group, a new study group or panel was recruited and the commencement of the study began with a posting to the group inviting participation in the survey. Sampling was completed for the online group in 9 days, while the paper-based group took 79 days. The time calculated included the registered completion of the survey notice via Fluid Surveys for the online group and the received postage or scanned return receipt date for the paper surveys. Factored into the calculation was the staff time, which included doing follow-up phone calls and emails to dietitians who were handling the in-person surveys, postage preparation, monitoring and tracking responses, and for recruitment of participants directly in the online condition.

## Discussion

### Principal Considerations and Findings

The many barriers to convening users, both logistically and methodologically, can impede health organizations’ willingness and capacity to engage in appropriate user testing of health materials. Rapid shifts in the way the public engages with information and information providers through modalities like Facebook, Twitter, and other social media channels has posed additional challenges for health professional organizations as the media cycle is shortened with consumers wanting materials quickly and expecting to have an opportunity to engage with the content materials in a more reflexive, interactive manner. ERO was already engaged with their users in conversations and exchanges via Facebook and thus, it provided a logical set-up for the study.

Other studies of recruitment have found that Facebook is a viable and cost-effective method for recruitment of study participants in different contexts [15]. Bensley et al found that clients are interested in using Web tools for nutritional information and recommended that Facebook be considered a key tool to support that work [16]. Lohse found that Facebook was an effective means of recruiting low-income women via ads distributed through the social media site [9]. This study differed from Lohse because it built on an established relationship with the intended users who had already indicated interest through responding to a request posted to the study hosts’ page, not an ad.

This study found no substantive differences in the nature and quality of feedback obtained through a survey delivered via a standard, in-person feedback process and one recruited through Facebook and deployed online. Further, the study demonstrated the feasibility of using social media as a recruitment tool and that comparable data could be collected through online methods to in-person methods. Statistically significant (.05) differences were detected on four attitudinal variables related to the document reviewed and include perceived length of the document, perceived attractiveness, likelihood of contacting ERO for food and nutrition questions in the future, and likelihood of recommending ERO to a friend. In all cases, the responses were more favorable to the document or ERO with the online group than the in-person group.

One of the differences detected between the two groups is with respect to the stated likelihood of using ERO materials or recommending its services (see Q10 and Q18 for specific item wording), with those in the online group reporting a greater likelihood to engage ERO further. This could be because the participants in the online group were recruited from an ERO-administered Facebook group, suggesting that prior interest in being connected to ERO could influence future considerations.

Another notable difference is that those in the online condition were more likely to suggest that the reviewed resource on healthy eating was “just right.” This could potentially suggest that the ability to touch, hold, and see the entire document in an in-person environment influences the perception of the design characteristics of that document. This is a question for further research to determine the degree to which the medium for soliciting feedback is connected to the product media used in communication.

### Limitations

This was an efficacy study aimed at exploring the feasibility and plausibility of using a virtual panel as a means of engaging users and soliciting quality, complete feedback in a comparative manner to the current standard of practice used by ERO. Although the study involved nutrition education materials developed by PEN, the focus was not to assess their educational impact, nor were the instruments used designed for such a purpose. The exploratory nature of the study and unknown possible outcomes meant that the sample size was low and thus the findings are not highly generalizable to other contexts. Further research will need to consider whether there are shifts in effects with greater numbers of participants.

Recruitment of participants through a Facebook group developed and administered by ERO could have introduced a positive bias to the responses to the survey. While the sample was non-random, both groups were drawn from consumers who fit the key demographics of ERO services on the measured variables. What the sample did not reflect was potential additional demographic groups, particularly those defined by cultural heritage, geographic location, and eHealth literacy or those in the community who were not able to or willing to engage community dietitians (for the in-person condition). Further research could explore whether these conditions are influential variables influencing the outcomes.

Another limitation is related to the population being researched and the gendered conditions in which most primary food decision making is being made, namely that women have been historically much more likely to engage with ERO than men in this role. Thus, it is unclear whether men would report similar things if equally represented in the sample. More research at a population level is needed now that the plausibility and efficacy has been explored through this study. While there are potential confounders that could influence the findings, the key is to understand that the overall viability and plausibility of comparing two different approaches and their implementation is sound even if the degree of impact of each method of response requires further study to delineate the full effects of each approach on the feedback received.

### Conclusions

This study sought to determine if the virtual panel approach is viable, to assess any unique challenges in the study implementation, and to propose strategies based on the findings to do further research with larger populations and different contexts. In doing so, the study also explored a methodological approach that could be used directly by health professionals and social marketing researchers. Our findings build on earlier research that showed how Facebook could be a cost-effective means of recruitment for participants. Indeed, ERO has since adopted this method in their work since the completion of the study and is using it as part of ongoing practice for feedback solicitation and is considerably increasing the frequency and speed of feedback in shaping the design of their materials. Consistent with the findings in this study, the use of an online panel has saved considerable resources in terms of coordination and energy required to reach and engage users.

Using online engagement methods for user testing has the further benefit of engaging current and prospective clients in dialogue early and potentially building a relationship with them in a manner that extends beyond a simple transactional encounter. By using tools like Facebook, participants are invited to be part of an initiative, not just participate in a study or survey. This creates additional value for potentially lower costs, which is an important advantage when there are limited financial and human resources for health communications.

By creating a means for greater engagement and a responsive method of feedback elicitation, health promotion organizers create opportunities to be more effective and relevant in their messaging. Social media provides opportunity to get feedback within days and through direct engagement with users instead of relying on intermediaries—a process that can introduce timing constraints. If approached as an opportunity to engage users in the design of products, social media offers means to speed up the process of and reducing the barriers to creating health promotion products and services that are not only attractive and useful, but more effective overall.

## Appendix

Multimedia Appendix 1 Healthy eating handout that was reviewed.

Multimedia Appendix 2 Data collection survey.

## Abbreviations

DC: Dietitians of Canada
ERO: EatRight Ontario
PEN: Practice-based Evidence in Nutrition
