# Supplementary material for: Exploring the Feasibility and Potential of Virtual Panels for Soliciting Feedback on Nutrition Education Materials: A Proof-of-Concept Study
Source: JMIR Public Health Surveill. 2016 Apr 19;2(1):e18. doi: 10.2196/publichealth.5134 (PMC4869232; doi:10.2196/publichealth.5134)
Supplement: Multimedia Appendix 1 [file publichealth_v2i1e18_app1.pdf]

EatRight Ontario

PEN The Global Resource for Nutrition Practice

Healthy Eating Guidelines for Increasing Your Fibre Intake

Why This Diet is Important

This fact sheet provides tips on how to add more fibre-rich foods in your diet. There are different kinds of fibre found in foods such as vegetables, fruit, legumes (peas, beans, lentils) and whole grains and cereals.

Some fibre can help to lower blood cholesterol and control blood glucose (sugar) levels. This is called soluble fibre and is found in some vegetables and fruit and legumes. Other types of fibre, called insoluble fibre from some vegetables and fruit, whole grains and wheat bran, help keep your bowels regular and may protect against colon cancer. Constipation may be improved with a diet that is high in fibre.

It is important to eat a variety of foods to get enough of both types of fibre. A healthy diet includes 25-38 grams of fibre a day. Talk to a dietitian if you want help on increasing the dietary fibre in your diet.

Steps you can take

Use Canada's Food Guide to plan your meals and snacks. Refer to Additional Resources below to get a copy of the guide

- Grains
  - Eat at least six servings of whole grain products every day. Examples include rolled oats, barley, brown rice, quinoa and whole wheat breads, breakfast cereals and pasta.
  - Use whole grain bread, rolls, pita or bagels when making toast or sandwiches.

© 2013 Dietitians of Canada. All rights reserved.
May be reproduced in its entirety provided source is acknowledged.

This information is not meant to replace advice from your medical doctor or individual counseling with a registered dietitian. It is intended for educational and informational purposes only.

- Replace at least half of the white flour with whole wheat flour in your recipes.
- Add 15-30 mL (1-2 Tbsp) of bran, ground flax seed or a very high-fibre cereal to your cereal in the morning.

- Vegetables and Fruit
  - Eat at least seven servings of vegetables and fruit every day. Aim for 1-2 servings of fruit and vegetables at every meal and snack.
  - Eat the peels of your vegetables and fruits whenever possible.
  - Try having fruit as a snack or as a dessert. Pears, raspberries and blackberries are top choices for fibre. Guava, persimmon and kumquat are exotic high fibre fruits worth trying.
  - Eat vegetables and fruit instead of drinking juice.
- Legumes
  - Add barley, beans, peas or lentils to soups, stews and casseroles. For example, add black beans to spaghetti sauce or lentils to soup.
  - Roast chickpeas or steam edemame (soybeans in a pod) for an easy snack.
  - Use legume-based dips (ex: hummus, baba ghanoush) for veggies instead of sour cream based.
- Nuts and Seeds
  - Add toasted nuts, sesame seeds, sunflower or pumpkin seeds to salads and cereals.
  - Sprinkle on pasta dishes and stir-fries.
  - Grab a handful of nuts (60 mL (1/4 cup) as a quick snack.
- Read Food Labels
  - Compare products and pick the one with the most fibre. Be sure you are comparing the same serving sizes.
  - Check for grams of fibre. High-fibre foods have four or more grams of fibre per serving.

© 2013 Dietitians of Canada. All rights reserved.
May be reproduced in its entirety provided source is acknowledged.

This information is not meant to replace advice from your medical doctor or individual counseling with a registered dietitian. It is intended for educational and informational purposes only.

- Look for ingredients such as bran, whole grain whole wheat, oatmeal or rye flour.
- Increase the fibre in your diet slowly to avoid gas, bloating and diarrhea.
  - Divide fibre-containing foods throughout the day at both meals and snacks.
  - Drink plenty of fluids.

Here is an example of how to increase your fibre intake with only a few small changes:

Low Fibre Diet	Fibre (g)	Fibre (g)	High Fibre Diet
Breakfast			
175 mL (3/4 cup) flaked corn cereal	0.8	5.0	125 mL (1/2 cup) flaked bran cereal
250 mL (1 cup) low fat milk	0.0	0.0	250 mL (1 cup) low fat milk
1 slice (35 g) white toast	0.9	3.2	1 slice (35 g) whole wheat toast
15 mL (1 Tbsp) jam	0.2	0.9	15 mL (1 Tbsp) peanut butter
125 mL (1/2 cup) orange juice	0.3	3.1	1 small orange
Lunch			
2 slices (70 g) white bread	1.8	6.2	2 slices (70g) whole wheat bread
75 g light tuna	0.0	0.0	75 g light tuna
15 ml (1 Tbsp) light mayonnaise	0.0	0.0	15 mL (1 Tbsp) light mayonnaise
1 medium leaf lettuce	0.1	0.1	1 medium leaf lettuce
1/2 tomato, sliced	0.8	0.8	½ tomato, sliced

© 2013 Dietitians of Canada. All rights reserved.
May be reproduced in its entirety provided source is acknowledged.

This information is not meant to replace advice from your medical doctor or individual counseling with a registered dietitian. It is intended for educational and informational purposes only.

125 mL (1/2 cup) vanilla yogurt	0.0	0.0	125 mL (1/2 cup) vanilla yogurt
		2.0	60 mL (1/4 cup) raspberries
Snack			
1 medium apple	2.6	5.0	1 medium pear
Water			Water
Supper			
75 g baked chicken breast	1.0	1.0	75 g baked chicken breast
125 mL (1/2 cup) mashed potato	1.6	4.0	1 medium baked potato with skin
125 mL (1/2 cup) carrots	2.2	2.2	125 mL (1/2 cup) carrots
250 mL (1 cup) low fat milk	0.0	1.2	250 mL (1 cup) romaine lettuce salad
125 mL (1/2 cup) vanilla pudding	0.0	0.0	250 mL (1 cup) low fat milk
		1.3	125 mL (1/2 cup) fruit cocktail
Snack			
4 melba toast crackers	1.3	2.5	1/2 whole wheat pita
30 g (1 oz) cheddar cheese	0.0	2.5	60 mL (1/4 cup) hummus
125 mL (1/2 cup) grapes	1.0	1.0	125 mL (1/2 cup) grapes
Water			Water
Total Fibre	15.8g	41.9g	

Information on fibre content is from the Canadian Nutrient File 2012.

© 2013 Dietitians of Canada. All rights reserved.
May be reproduced in its entirety provided source is acknowledged.

This information is not meant to replace advice from your medical doctor or individual counseling with a registered dietitian. It is intended for educational and informational purposes only.

Available from: http://webprod3.hc-sc.gc.ca/cnf-fce/newNutrientSearch-nouvelleRechercheAliment.do?lang=eng

Additional Resources

- Health Canada, Eating Well with Canada's Food Guide
  www.onpen.ca/Docviewer.aspx?id=1508
- EatRight Ontario, Food Sources of Fibre
  www.onpen.ca/Docviewer.aspx?id=7474

These resources are provided as sources of additional information believed to be reliable and accurate at the time of publication and should not be considered an endorsement of any information, service, product or company.

Notes

Distributed by EatRight Ontario:

Get answers to your nutrition and healthy eating questions from a place you can trust.

1. Call EatRight Ontario. Talk to a Registered Dietitian for FREE
   1-877-510-510-2, Monday – Friday 9 am to 5 pm ET.
   Evening hours Tuesday and Thursday to 9 pm ET.
2. Email a Dietitian from www.eatrightontario.ca.
3. Visit www.eatrightontario.ca

Dietitians of Canada acknowledges the financial support of EatRight Ontario by the Ontario government. The views expressed do not necessarily reflect those of the Province.

© 2013 Dietitians of Canada. All rights reserved.
May be reproduced in its entirety provided source is acknowledged.

This information is not meant to replace advice from your medical doctor or individual counseling with a registered dietitian. It is intended for educational and informational purposes only.
