# Supplementary material for: Exploring the Feasibility and Potential of Virtual Panels for Soliciting Feedback on Nutrition Education Materials: A Proof-of-Concept Study
Source: JMIR Public Health Surveill. 2016 Apr 19;2(1):e18. doi: 10.2196/publichealth.5134 (PMC4869232; doi:10.2196/publichealth.5134)
Supplement: Multimedia Appendix 2 [file publichealth_v2i1e18_app2.pdf]

Healthy Eating Handout:
A Study To Collect User Feedback

Introduction

Dietitians of Canada is seeking to improve their healthy eating materials and explore different ways to get client feedback on them.

This study is being conducted by Dr. Cameron Norman from the Dalla Lana School of Public Health at the University of Toronto working with the Dietitians of Canada. This study requires you to review a handout with information on healthy eating and complete a short survey with questions about what you think about this handout. The handout is attached to this survey.

After you have signed the consent form please proceed to the next page to complete the survey.

Dietitians of Canada
Les diététistes du Canada

EatRight Ontario
SPEAK WITH A REGISTERED DIETITIAN FOR FREE
1-877-510-510-2
www.eatrightontario.ca

UNIVERSITY OF TORONTO
DALLA LANA SCHOOL OF PUBLIC HEALTH

Healthy Eating Handout: A Study to Collect User Feedback

This study asks you to first read the handout, **Healthy Eating Guidelines for Increasing Your Fibre Intake**, and then answer the questions below.

Part 1: Layout and Design

This section will ask you questions on the look of the handout.

1. I find the size of the type (font):

   ☐ Too big
   ☐ Too small
   ☐ Just right

2. The handout length is:

   ☐ Too long
   ☐ Too short
   ☐ Just right

3. I think the handout is:

   ☐ Very attractive
   ☐ Somewhat attractive
   ☐ Not attractive
   ☐ Very unattractive

Additional comments on the look of the handout:

Part 2: Handout Content

This section will ask you questions about the content of the handout.

4. The amount of information I understand on the handout is:

☐ All
☐ Most
☐ Some
☐ None

5. The information provides me with helpful ways to change my diet:

☐ Strongly agree
☐ Agree
☐ Disagree
☐ Strongly disagree

6. I trust the information on the handout:

☐ Strongly agree
☐ Agree
☐ Disagree
☐ Strongly disagree

7. The handout did the following (Check all that apply):

☐ Confirmed what I already know about the topic
☐ Answered my questions about the topic
☐ Increased my knowledge on the topic
☐ Made me want to make changes to my diet
☐ Showed me that it is important to make changes to my diet
☐ Gave me the confidence that I could change my diet
☐ Helped me with ideas to change my diet
☐ None of the above

8. After reading the handout tell us 1-3 changes that you want to make to your diet:

Part 3: Additional Resources and Service Use

The next three questions look at the 'Additional Resources' section at the end of the handout.

9. I found the Additional Resources section was useful:

☐ Strongly Agree
☐ Agree
☐ Disagree
☐ Strongly Disagree

10. At the end of the handout there is a section that says "Distributed by EatRight Ontario".

If you are a resident of Ontario, how likely are you to contact EatRight Ontario for food and nutrition questions in the future?

☐ Very likely
☐ Somewhat likely
☐ Not likely
☐ Not at all likely
☐ I do not live in Ontario

11. How likely are you to recommend this handout to a friend?

☐ Very likely
☐ Somewhat likely
☐ Not likely
☐ Not at all likely

12. What would make this handout more useful to you?

One more page left...

Part 4: Information about you and using EatRight Ontario

The questions in this section will help us understand who is answering the survey and about your use of EatRight Ontario services.

13. My gender:

- ☐ Female
- ☐ Male
- ☐ Prefer not to answer

14. My age today:

- ☐ 13 or under
- ☐ 14-18
- ☐ 19-30
- ☐ 31-50
- ☐ 51-70
- ☐ 71 or over
- ☐ Prefer not to answer

15. The province I live in is:

____

16. The first three digits of my postal code are: ___________________

17. I have used the following EatRight Ontario services in the past two years

Check all that apply:

- ☐ Dietitian toll-free phone line service
- ☐ Email-a-Dietitian service
- ☐ The www.eatrightontario.ca website
- ☐ Follow @eatrightontario Twitter feed
- ☐ EatRight Ontario Facebook page
- ☐ EatRight Ontario YouTube videos
- ☐ eNewsletter
- ☐ I have not used any EatRight Ontario services in the last two years

18. How likely are you to recommend EatRight Ontario to a friend?

- ☐ Very likely
- ☐ Somewhat likely
- ☐ Not likely
- ☐ Not at all likely
- ☐ I do not live in Ontario

Thank you for completing this survey!
